# LQR-MPC-Based Trajectory-Tracking Controller of Autonomous Vehicle Subject to Coupling Effects and Driving State Uncertainties

**DOI:** 10.3390/s22155556

**Published:** 2022-07-25

**Authors:** Tengfei Yuan, Rongchen Zhao

**Affiliations:** School of Mechanical and Electrical Engineering, Guizhou Normal University, Guizhou 550025, China; yuantengfei@gznu.edu.cn

**Keywords:** trajectory tracking, lateral and longitudinal coupling control, Extended Kalman Filter (EKF) observer

## Abstract

This paper presents a lateral and longitudinal coupling controller for a trajectory-tracking control system. The proposed controller can simultaneously minimize lateral tracking deviation while tracking the desired trajectory and vehicle speed. Firstly, we propose a hierarchical control structure composed of upper and lower-level controllers. In the upper-level controller, the linear quadratic regulator (LQR) controller is designed to compute the desired front wheel steering angle for minimizing the lateral tracking deviation, and the model-predictive controller is developed to compute the desired acceleration for maintaining the planed vehicle speed. The lower-level controller enables the achievement of the desired steering angle and acceleration via the corresponding component devices. Furthermore, an observer based on the Extended Kalman Filter (EKF) is proposed to update the vehicle driving states, which are sensitive to the trajectory-tracking control and difficult to measure directly using the existing vehicle sensors. Finally, the Co-simulation (CarSim-MATLAB/Simulink) results demonstrate that the proposed coupling controller is able to robustly realize the trajectory tracking control and can effectively reduce the lateral tracking error.

## 1. Introduction

Autonomous vehicle technology is undoubtedly one of the hottest topics at the moment. Vehicular autonomy can be achieved in three main steps: the vehicle’s perception, its trajectory planning, and its control. The vehicle control system, that is, the trajectory-tracking control system, is the most important part of autonomous driving. The main goal for a trajectory-tracking control system is to design a control system that consists of lateral and longitudinal control modules and can perform the task of trajectory tracking for an autonomous vehicle. Indeed, the trajectory-tracking process of autonomous vehicles is coordinated by the lateral and longitudinal motion systems with complex coupling relationships, which should be taken into account when designing a trajectory-tracking controller [1,2]. Thus, the determination of a method to address the problem of controlling the lateral and the longitudinal vehicle dynamics in a coupled way remains a challenging task.

In the past few years, numerous control techniques have been studied to address the problem of trajectory tracking in autonomous vehicles. The existing control methods, such as sliding mode control (SMC) [3,4,5], robust control [6], model predictive control (MPC) [7,8,9,10,11,12], the linear quadratic regulator (LQR) [13,14,15,16,17,18,19,20,21], and the classic PID control [8,19], were proposed to pursue the task of lateral and longitudinal control. However, most of these studies aimed to address the lateral and longitudinal control separately. Recently, some researchers have been dedicated to addressing lateral and longitudinal vehicle dynamics control in a coupled way. Specifically, the authors of [20] proposed a coupling lateral and longitudinal control approach based on the SMC technique. However, the SMC technique usually results in chattering phenomenon while obtaining robustness. To cope with this drawback, the authors of [3] proposed an adaptive SMC approach based on lateral deviation, where an adjustable parameter related to the sliding surface and system error was introduced to reduce the chatter. However, the existence of system inertia will cause the switching delay of the SMC system, which is negative for vehicle’s lateral control.

The MPC and its derivative algorithms have been extensively investigated with respect to trajectory-tracking control because of its ability to deal with multiple constraints and nonlinear dynamics. For instance, the authors of [10] presented a multi-input–multi-output linear MPC approach that calculates the steering angle and the angular velocity of the vehicle to track the desired path by considering the vehicle dynamics constraint. Reference [7] proposed a novel MPC approach to force the vehicle to track the desired vehicle speed, and the non-PDC controller and the Lyapunov theorem were proposed to guarantee the stabilization. However, the constraints of acceleration and acceleration increment were not described. The authors of [8] adopted the Nonlinear model predictive control (NMPC) approach to solve the nonlinear muti-input–muti-output problem to achieve the lateral stability of the vehicle. However, with the increasing complexity of models and constrains, the huge amount of computation required by MPC is not conductive to real-time operations. Recently, the LQR controller based on a comprehensive lateral and longitudinal dynamics model was proposed in [18] to achieve a path-tracking task by controlling the steering angle and the torque applied to the wheels. However, only the braking performance was analyzed, and the results of the speed tracking were not given. The robust control approach has been compared with LQR and MPC in [6], which proved that the LQR has a better path-tracking performance in a parking scenario. Therefore, compared with MPC, the LQR technique requires less computation while considering multiple performance indicators, and in some cases has similar control performance to MPC. Motived by the analysis of the abovementioned works in the literature, the LQR should be considered as a lateral controller for autonomous vehicles. In addition, the LQR and MPC should be combined to design a novel trajectory-tracking controller that can consider both accuracy and real time.

Furthermore, the uncertainty of the vehicle states, such as the longitudinal velocity and yaw rate, are neglected in the above research. Indeed, considering the influence of the complex driving environment, some vehicle driving states are sensitive to the trajectory-tracking control, and are hard to measure directly using vehicle sensors. Thus, an online estimation approach for the trajectory-tracking controller should be designed to update these uncertainty states. The main approaches to the estimation of vehicle states are the Kalman Filter (KF) and its extended algorithms, such as the Extended Kalman Filter (EKF) and Unscented Kalman Filter (UKF), etc. [21]. In [22], the MPC controller was applied to the trajectory-tracking control system and the UKF was used to estimate the vehicle states. Reference [23] combined the EKF to obtain a higher vehicle state estimation accuracy. In practice, both the EKF and UKF can effectively address the state estimation of nonlinear systems [24,25], but the UKF uses the nonlinear equations to directly build the state space model, and the Unscented Transform (UT) is used to deal with the nonlinear propagate of the state mean and covariance matrix, while the EKF makes a linearization by ignoring the higher order terms of the expansion. Thus, the UKF has a slight improvement in accuracy than the EKF. However, the EKF is more computationally efficient than the UKF. For instance, the authors of [26] have found, after many experiments, that the UKF performed equivalently to the EKF; however, the EKF is generally faster than the UKF, and the fastest observer in the literature was about 2.6 times faster than the slowest observer. Therefore, combining the EKF observer with the controller for the trajectory-tracking control system that needs to deal with the strict real-time requirement is an ideal approach.

From the above literature survey, it has been proven that considering the coupling relationships between the lateral and longitudinal motion can effectively improve the trajectory tracking accuracy. However, most of the control approaches focus on the lateral and longitudinal control, while the literature on the lateral and longitudinal coupling control approach are not mature. Additionally, it is worth mentioning that to verify the control algorithm, most researchers usually assume that the vehicle states measured by the vehicle sensors are ideal values, which will affect the final control effect of the algorithm.

Inspired by the above discussion and considerations, this paper presents a trajectory-tracking control approach under the consideration of vehicle states’ uncertainty and lateral and longitudinal coupling relationships. The novelties and contributions of this paper are summarized as follows:

- A trajectory-tracking controller, which considers the coupling relationships of lateral and longitudinal dynamics for autonomous vehicles, is designed, guaranteeing: (i) the vehicle enables the tracking of the desired trajectory, and (ii) the speed error and lateral position error are within a small range;
- A vehicle driving state observer based on the EKF is proposed to update the vehicle driving states, such as longitudinal velocity and yaw rate, which are sensitive to the trajectory-tracking control and hard to measure directly using the existing vehicle sensors;
- Tests in a co-simulation (CarSim-MATLAB/Simulink) environment is presented to validate the effectiveness of the proposed controller.

With respect to the existing literature, the main merits of the proposed trajectory-tracking control approach are as follows. Compared with [16], this paper considers the coupling relationships between lateral and longitudinal dynamics and designs a longitudinal controller to track the planned vehicle speed. Compared with the path tracking approach presented in [10], the designed controller is tested in a wider speed range (30–118 km/h). Moreover, compared with the longitudinal control approach presented in [7,9], an EKF observer is established to estimate the longitudinal velocity, which is sensitive to the control process and is hard to measure directly.

The remainder of this paper is organized as follows. In Section 2, the framework of the proposed trajectory-tracking control system and the notation applied throughout this paper are described. In Section 3, a series of mathematical models are developed for the controller’s design: the lateral and longitudinal coupling control system model and the vehicle state estimation system model. A coupling controller for a trajectory-tracking control system is designed in Section 4. To demonstrate the efficiency of the proposed controller, the quintic polynomial trajectory planning approach is proposed to generate the desired trajectory and the desired vehicle speed in Section 5, while the co-simulation results are presented and analyzed. Finally, Section 6 summarizes the contents of this paper and describes the future work.

## 2. Framework of Trajectory-Tracking Control System

**Figure 1:**
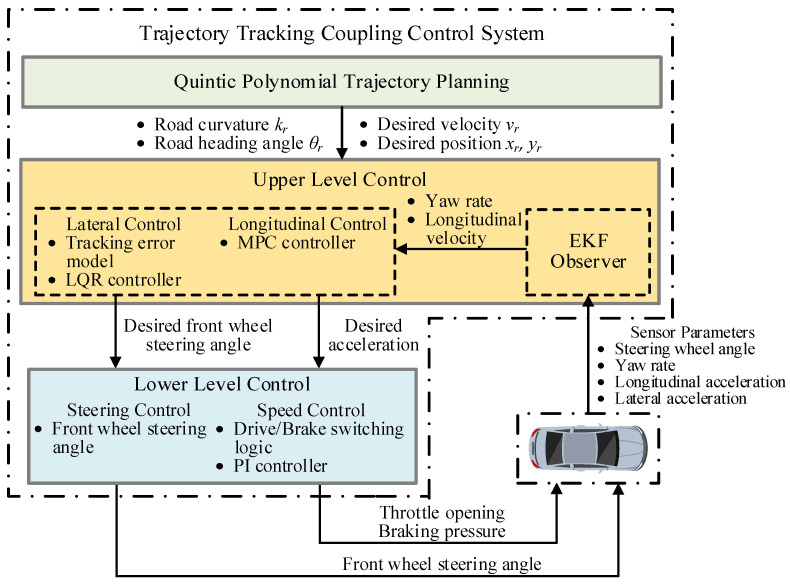
Hierarchical control structure of the proposed trajectory-tracking control system.

A hierarchical control structure is proposed to construct the lateral and longitudinal coupling controller for a trajectory-tracking control system, as shown in Figure 1. The overall target of the control system is to compute the desired front wheel steering angle and the desired acceleration and implement this target via the corresponding component devices to consequently track the desired trajectory. The hierarchical control structure is composed of an upper-level controller and a lower-level controller. The upper-level controller is designed to compute the desired front wheel steering angle and the desired acceleration. The EKF observer is established to obtain the longitudinal velocity and yaw rate, which are uncertain due to the coupling relationships and are hard to measure directly using the existing vehicle sensors. The lower-level controller achieves the output of the upper-level controller via the steering and driving devices to track the desired trajectory.

For the reader’s reference, Table 1 summarizes the main symbols and their corresponding descriptions that appear in this paper.

## 3. Trajectory-Tracking Control System Modeling

**Figure 2:**
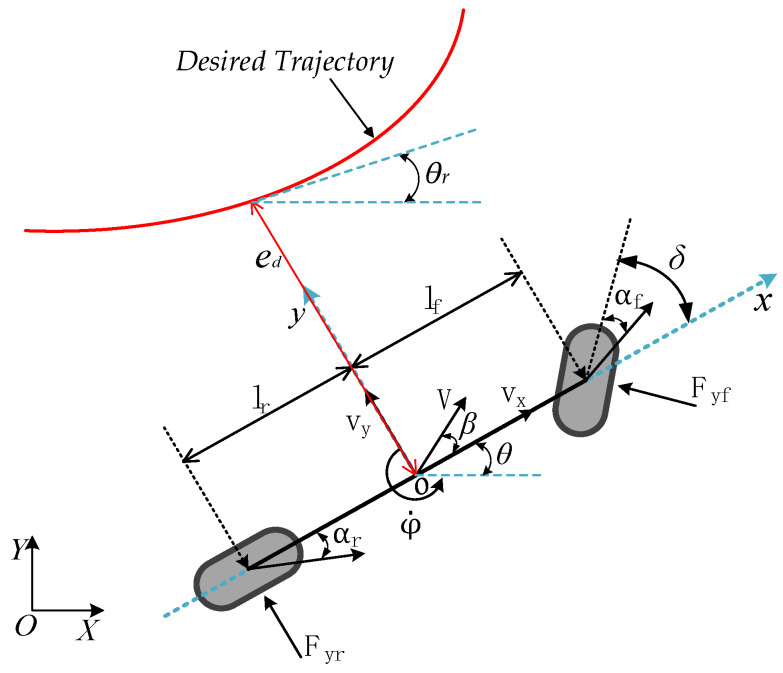
Vehicle dynamics model.

Overly complex vehicle models will occupy more computing resources and cause the control problem to be harder to solve. Thus, the real-time control algorithm can not meet the needs of real vehicle applications [27,28]. Therefore, in this section, considering the influence of the lateral motion, yaw motion, and longitudinal velocity of the vehicle, a simplified vehicle dynamics model that can reflect the vehicle motion characteristics as far as possible by means of constraint simplification and approximation is described, as shown in Figure 2.

The model is established based on the following assumptions:

- The vehicle runs on a flat road; that is, the vertical movement of the vehicle is not considered;
- The influence of the vehicle suspension system is not considered;
- The steering angle acts directly on the front wheels of the vehicle (Ackermann drive);
- The tires are in good contact with the ground;
- Load transfer is ignored;
- Air resistance is ignored.

By analyzing Newton’s second law and the torque balance equation, the dynamics differential equation of the vehicle can be obtained as follows:

(1) $$m\left({\dot{v}}_{y}+v_{x}\dot{\varphi }\right)=F_{yf}cos\delta +F_{yr},$$

(2) $$I_{z}\ddot{\varphi }=F_{yf}cos\delta l_{f}-F_{yr}l_{r},$$

(3) $${\dot{v}}_{x}=v_{y}\dot{\varphi }+a_{x}.$$

By analyzing the 3-DOF vehicle dynamics model, assuming that the front wheel steering angle is small, the lateral force of the tire is equal to the product of the cornering stiffness and the slip angle of the tire [28], and by setting $v_{y}=\dot{y}$, the dynamics differential equation can be obtained as follows:

(4) $$\left\{ \begin{array}{l} \ddot{y}=\frac{c_{af}+c_{ar}}{mv_{x}}\dot{y}+\left(\frac{l_{f}c_{af}-l_{r}c_{ar}}{mv_{x}}-v_{x}\right)\dot{\varphi }-\frac{c_{af}}{m}\delta \\ \ddot{\varphi }=\frac{l_{f}c_{af}-l_{r}c_{ar}}{l_{z}v_{x}}\dot{y}+\frac{{l_{f}}^{2}c_{af}+{l_{r}}^{2}c_{ar}}{l_{z}v_{x}}\dot{\varphi }+\frac{l_{f}c_{af}}{I_{z}}\delta \\ {\dot{v}}_{x}=\dot{y}\dot{\varphi }+a_{x} \end{array},\right.$$

where m is the mass of the vehicle; $l_{f}$ and $l_{r}$ are the front and rear wheelbases of the vehicle, respectively; $v_{x}$ and $v_{y}$ represent the velocity components of the velocity V at the center of the vehicle’s mass along the X and Y axis, respectively; $a_{x}$ is the longitudinal acceleration of the vehicle; φ is the yaw angle of the vehicle; $\dot{\varphi }$ is the yaw rate of the vehicle; β is the slip angle of the center of mass, which denotes the angle of the current velocity of the center of mass with respect to the longitudinal axis of the car; δ is the front wheels’ steering angle; $\alpha_{f}$ and $\alpha_{r}$ represent the slip angles of the front and rear tires, respectively; $C_{af}$ and $C_{ar}$ represent the cornering stiffness of the front and rear tires, respectively; $F_{yf}$ and $F_{yr}$ represent the lateral forces of the front and rear tires, respectively; and $I_{z}$ is the moment of inertia of the vehicle around the Z axis.

The intelligent vehicle will have errors in the process of tracking the desired trajectory, as shown in Figure 2. Defining the distance between the vehicle’s centroid and the projection point of the desired trajectory centerline as the lateral distance error $e_{d}$, and the deviation between vehicle and desired trajectory direction as the heading error $e_{\varphi }$, this tracking error can be measured and minimized.

The heading error is defined as $\varphi +\beta -\theta_{r}$ [28]. Since the value of the slip angle β is so small, by setting $e_{\varphi }=\varphi -\theta_{r}$ as the approximate heading error (this approximation is explained in Section 4.2), ${\dot{e}}_{\varphi }$ can be obtained:

(5) $${\dot{e}}_{\varphi }=\dot{\varphi }-{\dot{\theta }}_{r},$$

where $\theta_{r}$ is the heading angle at the current time for the desired trajectory.

By analyzing the vehicle dynamics model and supposing that the value of $\varphi -\theta_{r}$ is small, ${\dot{e}}_{d}$ can be obtained:

(6) $${\dot{e}}_{d}=\dot{y}+v_{x}e_{\varphi },$$

where $\dot{y}=Vsin\beta$ and $v_{x}=Vcos\beta$.

Substituting Equations (6) and (5) into Equation (4), ${\ddot{e}}_{d}$ and ${\ddot{e}}_{\varphi }$ can be obtained:

(7) $${\ddot{e}}_{d}=\left(\frac{C_{af}+C_{ar}}{mv_{x}}\right){\dot{e}}_{d}+\left(-\frac{C_{af}+C_{ar}}{m}\right)e_{\varphi }+\left(\frac{l_{f}C_{af}-l_{r}C_{ar}}{mv_{x}}\right){\dot{e}}_{\varphi }+\left(-\frac{C_{af}}{m}\right)\delta +\left(\frac{l_{f}C_{af}-l_{r}C_{ar}}{mv_{x}}-v_{x}\right){\dot{\theta }}_{r},$$

(8) $${\ddot{e}}_{\varphi }=\ddot{\varphi }-{\ddot{\theta }}_{r}.$$

Since the curvature of the road is usually gentle, the second derivative of $\theta_{r}$ is ignored, and so ${\ddot{e}}_{\varphi }$ can be simplified to:

(9) $${\ddot{e}}_{\varphi }=\ddot{\varphi }.$$

By writing Equations (5)–(7) and (9) in matrix form, the state space equation of the lateral tracking error of the vehicle can be obtained as follows:

(10) $$\left[\begin{array}{c} {\dot{e}}_{d}\\ {\ddot{e}}_{d}\\ {\dot{e}}_{\varphi }\\ {\ddot{e}}_{\varphi } \end{array}\right]=\left[\begin{array}{cccc} 0 & 1 & 0 & 0\\ 0 & \frac{c_{af}+c_{ar}}{mv_{x}} & -\frac{c_{af}+c_{ar}}{m} & \frac{l_{f}c_{af}-l_{r}c_{ar}}{mv_{x}}\\ 0 & 0 & 0 & 1\\ 0 & \frac{l_{f}c_{af}-l_{r}c_{ar}}{I_{z}v_{x}} & -\frac{l_{f}c_{af}-l_{r}c_{ar}}{l_{z}} & \frac{l_{f}^{2}c_{af}+l_{r}^{2}c_{ar}}{l_{z}v_{x}} \end{array}\right]\left[\begin{array}{c} e_{d}\\ {\dot{e}}_{d}\\ e_{\varphi }\\ {\dot{e}}_{\varphi } \end{array}\right]+\left[\begin{array}{c} 0\\ -\frac{c_{af}}{m}\\ 0\\ -\frac{l_{f}c_{af}}{I_{z}} \end{array}\right]\delta +\left[\begin{array}{c} 0\\ \frac{l_{f}c_{af}-l_{r}c_{ar}}{mv_{x}}-v_{x}\\ 0\\ \frac{l_{f}^{2}c_{af}+l_{r}^{2}c_{ar}}{I_{z}v_{x}} \end{array}\right]{\dot{\theta }}_{r}.$$

As with many previous studies [12,28,29], the longitudinal control of the vehicle can be represented by a first-order inertial system:

(11) $$\dot{a}=\frac{K_{s}}{\tau_{d}}\left(a_{des}-a\right),$$

where $K_{s}=1$ is the gain of system, $\tau_{d}$ is the time constant, $a_{des}$ is the desired acceleration, and a is the vehicle’s actual acceleration.

The state equation for longitudinal motion can be expressed as:

(12) $$\dot{\xi }=A_{L}\xi +B_{L}u_{L},$$

where $\xi ={\left[V,a\right]}^{T}$ is the system state, V is the speed of the center of vehicle mass but also the control output of the system, and $u_{L}=a_{des}$ is the control input.

(13) $$A_{L}=\left[\begin{array}{cc} 0 & 1\\ 0 & -\frac{1}{\tau_{d}} \end{array}\right],\;B_{L}=\left[\begin{array}{c} 0\\ \frac{K_{s}}{\tau_{d}} \end{array}\right].$$

The lateral and longitudinal controllers have been modeled as shown in Equations (10)–(12). Thus, the linear time-varying model for the lateral and longitudinal coupling controllers can be established as follows:

(14) $$\dot{\chi }=\phi_{t}\chi_{t}+\eta_{t}u_{i,t}+\gamma_{t}{\dot{\theta }}_{r},$$

where $\chi_{t}={\left[e_{d},{\dot{e}}_{d},e_{\varphi },{\dot{e}}_{\varphi },V,a\right]}^{T}$ is the system state, and $u_{i,t}={\left[\delta ,a_{des}\right]}^{T}$ is the control input.

(15) $$\begin{array}{rl} & \phi_{t}=\left[\begin{array}{cccc} 0 & 1 & 0 & 0\\ 0 & \frac{c_{af}+c_{ar}}{mv_{x}} & -\frac{c_{af}+c_{ar}}{m} & \frac{l_{f}c_{af}-l_{f}c_{ar}}{mv_{x}}\\ 0 & 0 & 0 & 1\\ 0 & \frac{l_{f}c_{af}-l_{r}c_{ar}}{I_{z}v_{x}} & -\frac{l_{f}c_{af}-l_{r}c_{ar}}{I_{z}} & \frac{l_{f}^{2}c_{af}+l_{r}^{2}c_{ar}}{I_{z}v_{x}}\\ 0 & 0 & 0 & 1\\ 0 & 0 & 0 & -\frac{1}{\tau_{d}} \end{array}\right],\;\eta_{t}=\left[\begin{array}{cc} 0 & 0\\ -\frac{c_{af}}{m} & 0\\ 0 & 0\\ -\frac{l_{f}c_{af}}{I_{z}} & 0\\ 0 & \frac{ks}{\tau_{d}} \end{array}\right],\\ & \gamma_{t}=\left[\begin{array}{c} \frac{l_{f}C_{af}-l_{r}C_{ar}}{mv_{x}}-v_{x}\\ 0\\ \frac{l_{f}^{2}C_{af}+l_{r}^{2}C_{ar}}{I_{z}v_{x}}\\ 0\\ 0 \end{array}\right]. \end{array}$$

## 4. Coupling Controller Design

### 4.1. Control Objectives

In this work, we aim to propose a controller that can track the desired trajectory and the speed by considering the coupling relationships between the vehicle’s lateral and longitudinal dynamics. Therefore, aiming to ensure the accurate tracking to the time-varying planning trajectory, the speed and the lateral position of the vehicle should be controlled. The targets of the coupling controller can be considered as:

(16) $$Objective\left(A\right):V\to v_{r},$$

(17) $$Objective\left(B\right):e_{d}\to 0,$$

where $v_{r}$ is the planning speed; V is the speed at the center of vehicle mass, and when the vehicle reaches the steady state, the value of V should approach $v_{r}$ and the error should be as small as possible; $e_{d}$ denotes the lateral position error.

The objective of longitudinal control is to track the planning speed to improve the lateral tracking accuracy. At this point, the first objective of the coupling controller can be expressed as Equation (16).

The trajectory-tracking controller ensures that the vehicle travels along the planned trajectory. Therefore, another objective is to track the desired trajectory and minimize the lateral position error, and it can be expressed as Equation (17).

In addition, to track the planning speed while achieving ride comfort, some physical characteristics of the vehicle should also be considered. Thus, the acceleration and the acceleration increment should be constrained as:

(18) $$\{\begin{array}{c} a_{L,min}\leq a_{L}\leq a_{L,max},\;\;\;\;\;\;\;\;\\ \Delta a_{L,min}\leq \Delta a_{L}\leq \Delta a_{L,max}, \end{array},$$

where $a_{L,min}$, $a_{L,max}$, $\Delta a_{L,min}$, and $\Delta a_{L,max}$ are the bounds of the acceleration and the acceleration increment.

### 4.2. Upper Level Controller Design

Since longitudinal velocity is time-varying, Equation (10) is rewritten as a linear time-varying system as follows:

(19) $$\dot{X}=A_{t}X_{t}+Bu_{t}+C_{t}{\dot{\theta }}_{r}.$$

The controller is used in the discrete-time model. Thus, the continuous system needs to be converted into a discrete system. Ignoring the influence of term $C_{t}{\dot{\theta }}_{r}$, by using the midpoint Euler method and the forward Euler method on Equation (19) and simplifying it, the discretization model for the lateral tracking error can be obtained as follows:

(20) $$X_{k+1}={\bar{A}}_{k}X_{k}+\bar{B}u_{k},$$

where

(21) $${\bar{A}}_{k}={\left(I-\frac{A_{t}}{2}dt\right)}^{-1}\left(I+\frac{A_{t}}{2}dt\right),\;\bar{B}=Bdt.$$

The optimal control performance function for LQR is established as follows:

(22) $$J=\sum_{k=0}^{\infty }\left(X_{k}{}^{T}QX_{k}+u_{k}{}^{T}Ru_{k}\right),$$

where $X_{k}$ is the state variable of the system, $u_{k}$ is the control variable of the system, and Q and R are the weighting matrices of the state error and the control quantity, respectively. According to several experiments, the weighting matrices must be set to  Q=diag[28,1,4,1]  and R=10. When J is minimum, the control quantity u is the desired front wheel steering angle.

Substituting Equation (20) into (22) as a constraint, the Lagrangian control problem with multiplicative constraints is constructed as follows:

(23) $$J=\sum_{k=0}^{n-1}\left[X_{k}{}^{T}QX_{k}+u_{k}{}^{T}Ru_{k}+\lambda_{k+1}^{T}\left({\bar{A}}_{k}X_{k}+\bar{B}u_{k}\right)-\lambda_{k+1}^{T}X_{k+1}\right]+X_{n}{}^{T}QX_{n}.$$

Now, the Hamiltonian function can be constructed as follows:

(24) $$H_{k}=X_{k}{}^{T}QX_{k}+u_{k}{}^{T}Ru_{k}+\lambda_{k+1}^{T}\left({\bar{A}}_{k}X_{k}+\bar{B}u_{k}\right).$$

Substituting Equation (24) into (23) and simplification obtains:

(25) $$J=\sum_{k=0}^{n-1}\left[H_{k}-\lambda_{k}^{T}X_{k}\right]+X_{n}{}^{T}QX_{n}+\lambda_{0}{}^{T}X_{0}-\lambda_{n}{}^{T}X_{n}.$$

Now, the extreme value of Equation (25) can be obtained as follows:

(26) $$u_{k}=-{\left(R+{\bar{B}}^{T}P_{k+1}\bar{B}\right)}^{-1}{\bar{B}}^{T}P_{k+1}{\bar{A}}_{k}X_{k},$$

where $P_{k+1}$ is the solution of Riccati equation $P=Q+{\bar{A}}_{k}{}^{T}P{\bar{A}}_{k}-{\bar{A}}_{k}{}^{T}P\bar{B}{\left(R+{\bar{B}}^{T}P\bar{B}\right)}^{-1}{\bar{B}}^{T}P{\bar{A}}_{k}$.

Setting $\;K={\left(R+{\bar{B}}^{T}P_{k+1}\bar{B}\right)}^{-1}{\bar{B}}^{T}P_{k+1}{\bar{A}}_{k}$, the control quantity of the LQR controller can be obtained as follows:

(27) $$u_{k}=-KX_{k},$$

where $\;K=\left[k_{1},k_{2},k_{3},k_{4}\right]$ is the gain of the LQR controller.

Substituting Equation (27) into (19) obtains:

(28) $$\dot{X}=\left(A_{k}-BK\right)X_{k}+C_{k}{\dot{\theta }}_{r}.$$

According to the above equation, no matter what the value of gain K is, the distance error and heading error of the intelligent vehicle cannot be guaranteed to be zero in the control process; that is, there is a steady-state error in the system. Hence, in this paper, the feedforward controller based on feedback is designed to eliminate the steady-state error.

The actual control quantity after introducing feedforward $\delta_{f}$ is:

(29) $$u_{k}=-KX_{k}+\delta_{f}.$$

Substitute Equation (29) into (19) so that $\dot{X}=0$; that is, assume that the system reaches a steady state where the steady-state error is:

(30) $$X=-{\left(A_{k}-BK\right)}^{-1}\left(B\delta_{f}+C_{k}{\dot{\theta }}_{r}\right).$$

By solving Equation (30) and simplifying it, the following can be obtained:

(31) $$\left[\begin{array}{c} \begin{array}{c} e_{d}\\ {\dot{e}}_{d}\\ e_{\varphi } \end{array}\\ {\dot{e}}_{\varphi } \end{array}\right]=\left[\begin{array}{c} -\frac{1}{k_{1}}\{\delta_{f}-\frac{{\dot{\theta }}_{r}}{vx}\left[l_{f}+l_{r}-l_{r}k_{3}-\frac{mv_{x}^{2}}{l_{f}+l_{r}}\left(\frac{l_{r}}{C_{af}}+\frac{l_{f}}{C_{ar}}k_{3}-\frac{l_{f}}{C_{ar}}\right)\right]\}\\ 0\\ \begin{array}{c} -\frac{{\dot{\theta }}_{r}}{vx}\left(l_{r}+\frac{l_{f}}{l_{f}+l_{r}}\frac{mv_{x}^{2}}{C_{ar}}\right)\\ 0 \end{array} \end{array}\right].$$

The analytic Equation (31) shows that when the lateral distance error $e_{d}=0$, the feedforward control quantity $\delta_{f}$ is:

(32) $$\delta_{f}=-\frac{{\dot{\theta }}_{r}}{vx}\left[l_{f}+l_{r}-l_{r}k_{3}-\frac{mv_{x}^{2}}{l_{f}+l_{r}}\left(\frac{l_{r}}{C_{af}}+\frac{l_{f}}{C_{ar}}k_{3}-\frac{l_{f}}{C_{ar}}\right)\right].$$

In Equation (5), the actual heading error is assumed to be $e_{\varphi }=\varphi -\theta_{r}$ for ease of calculation. When the vehicle reaches the steady state, it is necessary to make the heading error $e_{\theta }=0$, and there is $e_{\varphi }=\varphi -\theta_{r}=-\beta$. Thus, it is not necessary to design a feedforward controller to eliminate the steady-state error of $e_{\varphi }$. Meanwhile, the authors of [30] proved that the steady-state equilibrium can still be achieved where the values of lateral error and heading angle error are nonzero.

The proposed MPC is a discrete-time strategy, and the continuous system should be discretized.

By using the forward Euler method on Equation (12) and simplifying it, the discretization model for a longitudinal control system can be obtained as follows:

(33) $$\xi_{k+1}={\bar{A}}_{L,k}\xi_{k}+{\bar{B}}_{L,k}u_{L,k},$$

where

(34) $${\bar{A}}_{L,k}=\left[\begin{array}{cc} 1 & 0\\ 0 & 1-\frac{T_{s}}{\tau_{d}} \end{array}\right],{\bar{B}}_{L,k}=\left[\begin{array}{c} 0\\ \frac{K_{s}T_{s}}{\tau_{d}} \end{array}\right],$$

where $T_{s}$ is the sampling period.

The output equation of the longitudinal control system can be expressed as:

(35) $$\Pi_{k}=\left[1,0\right]\xi_{k}.$$

We next track the desired speed accurately and smoothly by penalizing acceleration or excessive changes in acceleration. Formulating the control problem with a cost function:

(36) $$J_{s}\left(\xi_{k},u_{L,k-1},\Delta u_{L,k}\right)=\sum_{i=1}^{N_{p}}\|\Pi_{p,(k+i|k)}-\Pi_{ref,(k+i|k)}{\|}_{Q_{L}}^{2}+\sum_{i=0}^{N_{c-1}}\|\Delta u_{L,k+i}{\|}_{R_{L}}^{2},$$

where $N_{p}$ is the prediction time domain; $N_{c}$ is the control time domain; $\Pi_{p,(k+i|k)}\;$ is the prediction for the control output variable; $\Pi_{ref,(k+i|k)}$ is the reference for the control output variable; (k+i|k) represent the fact that the value at sampling time (k+i) is predicted based on the information from the sampling time k, where $i=1,\;2,\;\ldots ,\;N_{p}$; $Q_{L}$ is the weight matrix of the system output, reflecting the tracking accuracy of the control system to the reference velocity; and $R_{L}$ is the weight matrix of the system control increment.

Considering the comfort of the driver, the acceleration and the acceleration increment should be constrained, and the constraints can be formulated as follows:

(37) $$u_{L,min}\leq u_{L,k+i}\leq u_{L,max},\;i=0,1,\ldots N_{c}-1,\;\Delta u_{L,min}\leq \Delta u_{L,k+i}\leq \Delta u_{L,max},\;i=0,1,\ldots N_{c}-1,$$

where $u_{L,min}$ and $u_{L,max}$ are the minimum and maximum acceleration, respectively, and $\Delta u_{L,min}$ and $\Delta u_{L,max}$ are the minimum and maximum acceleration increment, respectively.

Notice that the longitudinal velocity and yaw rate are time-varying during the driving process. As presented in the introduction, the time-varying characteristic of the vehicle states is a critical issue in the trajectory-tracking controller design, so the EKF observer is designed to estimate them.

As can be seen from the geometric relationship in Figure 2, the slip angles of the front and rear tires and the slip angle of the centroid of the vehicle can be expressed as:

(38) $$\alpha_{f}=\beta +\frac{l_{f}\omega }{v_{x}}-\delta ,$$

(39) $$\alpha_{r}=\beta -\frac{l_{r}\omega }{v_{x}},$$

(40) $$\beta =\frac{v_{y}}{v_{x}}.$$

Through calculation and simplification in combination with Equation (4), the state equation and observation equation based on a 3-DOF nonlinear vehicle model can be obtained:

(41) $$\{\begin{array}{l} \dot{\omega }=\frac{l_{f}^{2}C_{af}+l_{r}^{2}C_{ar}}{I_{z}v_{x}}\omega +\frac{l_{f}C_{af}-l_{r}C_{ar}}{I_{z}}\beta -\frac{l_{f}C_{af}}{I_{z}}\delta \;\;\;\\ \dot{\beta }=\left(\frac{l_{f}C_{af}-l_{r}C_{ar}}{mv_{x}^{2}}-1\right)\omega +\frac{C_{af}+C_{ar}}{mv_{x}}\beta -\frac{C_{af}}{mv_{x}}\delta \\ {\dot{v}}_{x}=\omega \beta v_{x}+a_{x}\;\;\;\;\;\;\;\;\;\;\;\;\;\;\;\;\;\;\;\;\;\;\;\;\;\;\;\;\;\;\;\;\;\;\;\;\;\;\;\;\;\;\;\;\;\;\;\;\;\;\; \end{array},$$

(42) $$a_{y}=\frac{l_{f}C_{af}-l_{r}C_{ar}}{mv_{x}}\omega +\frac{C_{af}+C_{ar}}{m}\beta -\frac{C_{af}}{m}\delta ,$$

where $\omega =\dot{\varphi }$ is the yaw rate of the vehicle, $a_{x}$ is the longitudinal acceleration of the vehicle, and $a_{y}$ is the lateral acceleration of the vehicle.

The state Equation (41) and the observation Equation (42) are discretized to obtain:

(43) $$\left\{ \begin{array}{c} \omega (k)=\left[\frac{l_{f}^{2}C_{af}+l_{r}^{2}C_{ar}}{I_{z}v_{x}(k-1)}\omega (k-1)+\frac{l_{f}C_{af}-l_{r}C_{ar}}{I_{z}}\beta (k-1)-\frac{l_{f}C_{af}}{I_{z}}\delta (k-1)\right]\Delta t+\omega (k-1)\\ \beta (k)=\left[\left(\frac{l_{f}C_{af}-l_{r}C_{ar}}{m{\left[v_{x}(k-1)\right]}^{2}}-1\right)\omega (k-1)+\frac{C_{af}+C_{ar}}{mv_{x}(k-1)}\beta (k-1)-\frac{C_{af}}{mv_{x}(k-1)}\delta \right]\Delta t+\beta (k-1)\\ v_{x}(k)=\left[\omega (k-1)\beta (k-1)v_{x}(k-1)+a_{x}(k-1)\right]\Delta t+v_{x}(k-1) \end{array}\right.$$

(44) $$a_{y}\left(k-1\right)=\frac{l_{f}C_{af}-l_{r}C_{ar}}{mv_{x}\left(k-1\right)}\omega \left(k-1\right)+\frac{C_{af}+C_{ar}}{m}\beta \left(k-1\right)-\frac{C_{af}}{m}\delta \left(k-1\right).$$

Rewriting the discretized state equation and observation equation into the form of state space equation yields:

(45) $$\{\begin{array}{c} x\left(k\right)=f\left(x\left(k-1\right),u\left(k-1\right)\right)+W_{k}\\ y\left(k\right)=h\left(x\left(k-1\right),u\left(k-1\right)\right)+V_{k} \end{array},$$

where $x\left(k\right)={\left[\omega ,\beta ,v_{x}\right]}^{T}$ is the state variable, $u\left(k\right)={\left[\delta ,a_{x}\right]}^{T}$ is the input variable, and $a_{y}$ is the observation variable. $W_{k}$ and $V_{k}$ are the system noise and measurement noise, respectively, and they are independent of each other and their mean value is zero, the variance of $W_{k}$ is $Q_{k}$, and the variance of $V_{k}$ is $R_{k}$.

Then, the state equation and observation equation are linearized to obtain the Jacobian matrix:

(46) $$F=\frac{\partial F}{\partial x}=[\begin{array}{ccc} \frac{l_{f}^{2}c_{af}+l_{r}^{2}c_{ar}}{I_{z}v_{x}} & \frac{l_{f}c_{af}-l_{f}c_{ar}}{l_{z}} & -\frac{\left(l_{f}^{2}C_{af}+l_{r}^{2}C_{ar}\right)}{I_{z}v_{x}^{2}}\omega \\ \frac{l_{f}C_{af}-l_{r}C_{ar}}{mv_{x}^{2}}-1 & \frac{C_{af}+C_{ar}}{mv_{x}} & -\frac{2\left(l_{f}C_{af}-l_{r}C_{ar}\right)}{mv_{x}^{3}}\omega -\frac{C_{af}+C_{ar}}{mv_{x}^{2}}\beta +\frac{l_{f}\delta }{mv_{x}^{2}}\\ \beta v_{x} & \omega v_{x} & \omega \beta \end{array}],$$

(47) $$H=\frac{\partial h}{\partial x}=\left[\begin{array}{cc} \frac{l_{f}C_{af}-l_{r}C_{ar}}{mv_{x}} & \begin{array}{cc} \frac{C_{af}+C_{ar}}{m} & -\frac{l_{f}C_{af}-l_{r}C_{ar}}{mv_{x}^{2}} \end{array} \end{array}\right].$$

The Extended Kalman Filter consists of a prediction step and an update step.

1. The prediction step first predicts the state variable ${\hat{x}}_{k|k-1}$ at time *k* by the state variable ${\hat{x}}_{k-1}$ at time (*k* − 1):
  (48) $${\hat{x}}_{k|k-1}=f_{k-1}\left({\hat{x}}_{k-1},u_{k-1}\right).$$
  The prediction error covariance matrix $P_{k,\;k-1}$ is then computed:
  (49) $$P_{k,\;k-1}=FP_{k-1}F^{T}+Q_{k}.$$
2. In the update step, the state variable ${\hat{x}}_{k|k-1}$ is modified by Kalman gain $K_{l}$, and the state variable ${\hat{x}}_{k}$ at time *k* is then computed:
  (50) $${\hat{x}}_{k}={\hat{x}}_{k|k-1}+K_{l}\left[Y_{k}-h_{k}\left({\hat{x}}_{k|k-1},u_{k-1}\right)\right],$$
  where Kalman gain $K_{l}=P_{k,\;k-1}H^{T}{\left(HP_{k,\;k-1}H^{T}+R_{k}\right)}^{-1}$.

The state error covariance matrix $P_{k}$ is then computed:

(51) $$P_{k}=\left(I-K_{l}H\right)P_{k,k-1},$$

where $Q_{k}$ and $R_{k}$ are both Gaussian white noise with zero mean and independence.

For the EKF observer, the appropriate system noise covariance matrix $Q_{k}$ and measurement noise covariance matrix $R_{k}$ need to be selected, and the algorithm starts with the initial values $X_{0}$ and $P_{0}$. In this paper, we consider that $Q_{k}$ and $R_{k}$ are constants and that the setting of these parameters follows the method proposed by Schneider and Georgakis [31]. $Q_{k}$ and $R_{k}$ depend on the system and the sensors and should be determined by the user’s system and sensors. After constant debugging, we obtain two values: $Q_{k}=diag\left[1,1,1\right]$ and $R_{k}=1$. The initial value of the state error covariance matrix is set as $P_{0}=diag$ [0.001, 0.001, 0.001].

### 4.3. Lower Level Controller Design

**Figure 3:**
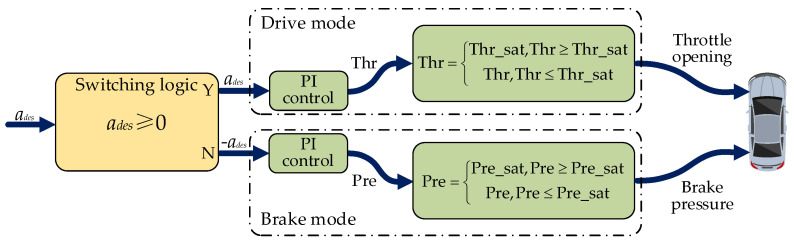
Lower-level control schematic for drive/brake system.

In order to validate the proposed control algorithm, a lower-level controller needs to be established to convert the output of the coupling controller into the input of the actuator of the simulated vehicle. Among them, the steering system’s control input is the front wheel steering angle, and the control inputs of the drive system are the throttle opening and brake master cylinder pressure. A control strategy is proposed to convert the desired acceleration from the coupling controller into the control input of the drive system. The control strategy consists of two parts: the switching logic of the brake and drive and the calculation of the actuator control input. The control schematic of the drive/brake system is defined as shown in Figure 3.

To prevent the vehicle from controlling the throttle pedal and the brake pedal simultaneously, it is necessary to design a switching logic for the drive and brake. In this paper, the switching of the drive and brake can be determined by comparing $a_{des}$ to zero, and if $a_{des}\geq 0$, the drive control is applied, otherwise the brake control is applied. The switching logic can be expressed as follows:

(52) $$a_{t}=\{\begin{array}{l} \begin{array}{cc} a_{des}, & a_{des}\geq 0 \end{array}\\ \begin{array}{cc} 0, & \;\;\;\;\;a_{des}<0 \end{array} \end{array},$$

(53) $$P_{b}=\{\begin{array}{l} \begin{array}{cc} 0, & \;\;\;\;\;\;\;\;\;a_{des}\geq 0 \end{array}\\ \begin{array}{cc} -a_{des}, & a_{des}<0 \end{array} \end{array},$$

where $a_{t}$ is the acceleration in drive mode while $P_{b}$ is the acceleration in brake mode.

A PI controller is applied to convert the desired acceleration into actuator control inputs, which are the desired throttle opening and the desired brake master cylinder pressure for the simulated vehicle platform. Considering the dynamic performance of the simulated vehicle, the desired throttle opening and brake master cylinder pressure are limited to a certain range. This paper restricts it as follows:

(54) $$Thr=\{\begin{array}{l} \begin{array}{cc} Thr\_sat, & Thr\geq Thr\_sat \end{array}\\ \begin{array}{cc} Thr, & \;\;\;\;\;\;\;\;Thr<Thr\_sat \end{array} \end{array},$$

(55) $$Pre=\{\begin{array}{l} \begin{array}{cc} Pre\_sat, & Pre\geq Pre\_sat \end{array}\\ \begin{array}{cc} Pre, & \;\;\;\;\;\;\;\;Pre<Pre\_sat \end{array} \end{array},$$

where Thr and Pre are desired throttle opening and brake master cylinder pressure, respectively, and Thr_sat=1 and Pre_sat=6 are the maximum throttle opening and brake master cylinder pressure, respectively.

## 5. Simulation Verification

To verify the effectiveness of the proposed controller, a co-simulation was performed with CarSim and MATLAB/Simulink, and the simulation results are compared with a classic MPC controller. The CarSim software delivers an accurate and efficient method for simulating the performance of a real vehicle [32]. The details of the simulation are described in the following subsections.

### 5.1. Trajectory Tracking Algorithm Verification

This subsection describes the approach of generating the desired trajectory for the trajectory-tracking controller, and three defferent simulation scenarios are designed to verify the effectiveness of the proposed controller.

#### 5.1.1. Desired Trajectory Setting

**Figure 4:**
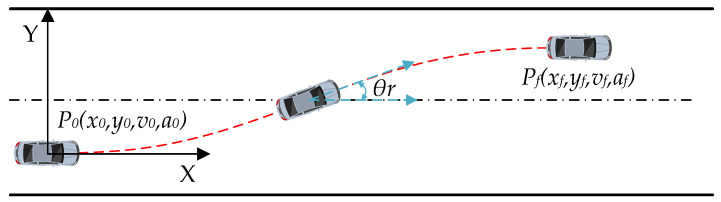
Schematic of lane change trajectory.

A quintic polynomial function can plan a trajectory that has a continuous third derivative and smooth curvature according to the initial and final states of the vehicle, so it is widely used in lane change scenarios [33]. As a result, this paper employs a time-based quintic polynomial function to generate the desired trajectory, and the effectiveness of the proposed coupling controller is verified by using a lane change scenario. The lane-change trajectory is shown in Figure 4. $P_{0}$ and $P_{f}$ present the initial position and the final position of the planning trajectory, respectively.

The quintic polynomial function can be described as:

(56) $$\{\begin{array}{c} x\left(t\right)=a_{0}+a_{1}t+a_{2}t^{2}+a_{3}t^{3}+a_{4}t^{4}+a_{5}t^{5}\;\;\\ y\left(x\right)=b_{0}+b_{1}x+b_{2}x^{2}+b_{3}x^{3}+b_{4}x^{4}+b_{5}x^{5} \end{array},$$

where $a_{0}$, $a_{1}$,..., $a_{5}$, $b_{0}$, $b_{1}$, …, $b_{5}$ are the designed parameters, which can be determined by the initial and final states of the vehicle.

The heading angle $\theta_{r}$ and the curvature $k_{r}$ for planning the trajectory can be computed by:

(57) $$\theta_{r}\left(t\right)=\arctan\left\{y^{\prime }\left[x\left(t\right)\right]\right\},$$

(58) $$k_{r}\left(t\right)=\frac{y^{\prime \prime }\left[x\left(t\right)\right]}{{\left(1+{\left\{y^{\prime }\left[x\left(t\right)\right]\right\}}^{2}\right)}^{\frac{3}{2}}}.$$

The velocity and acceleration constraints for planning the trajectory can be computed according to:

(59) $$v_{p}\left(t\right)=\sqrt{{\dot{x}}^{2}\left(t\right)+{\dot{y}}^{2}\left(t\right)},$$

(60) $$\{\begin{array}{c} a_{p}\left(t\right)=\sqrt{{\ddot{x}}^{2}\left(t\right)+{\ddot{y}}^{2}\left(t\right)},\;\;\;\;\;\;\;\;\;\;\;\;\ddot{x}\left(t\right)\geq 0;\\ a_{p}\left(t\right)=-\sqrt{{\ddot{x}}^{2}\left(t\right)+{\ddot{y}}^{2}\left(t\right)},\;\;\;\;\;\;\;\;\ddot{x}\left(t\right)\leq 0; \end{array}.$$

#### 5.1.2. Trajectory-Tracking Simulation Scenarios Setting

**Figure 5:**
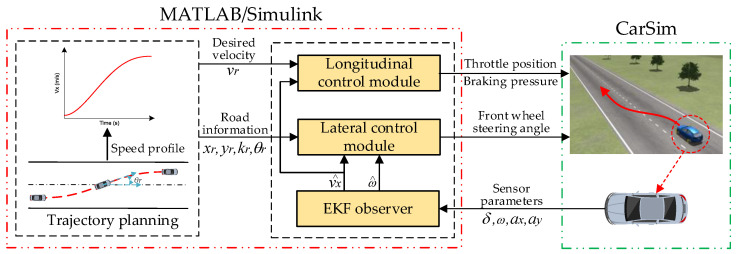
Co-simulation block diagram.

To verify the effectiveness of the proposed controller, a co-simulation was established in CarSim and MATLAB/Simulink environment. The block diagram of the co-simulation is shown in Figure 5. Three simulation scenarios are set (low speed, medium-high speed, and high speed) in this section. The main parameters for the co-simulation are shown in Table 2. It should be pointed out that D is the longitudinal movement distance in the process of lane change, W is the lane width, the tire-road friction factor f is 0.85, and the road environment is applied to all the simulation scenarios. In addition, the conventional MPC-based lateral and longitudinal coupling controller is provided for comparison.

In the first scenario, the simulation vehicle enters the lane changing trajectory with an initial velocity of 30 km/h, reaches the end of the planning trajectory with a final velocity of 40 km/h, and continues to travel at the final velocity. The other two simulation scenarios are similar to the first one; the detailed information of the three scenarios is shown in Table 3. Scenarios A, B, and C represent the first scenario, the second scenario, and the third scenario, respectively.

#### 5.1.3. Simulation Results and Analysis

**Figure 6:**
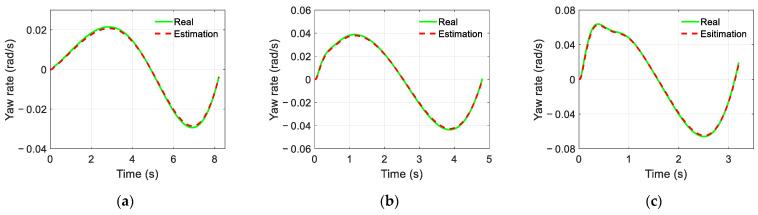
Yaw rate estimated by EKF: (**a**) Scenario A; (**b**) Scenario B; (**c**) Scenario C.

**Figure 7:**
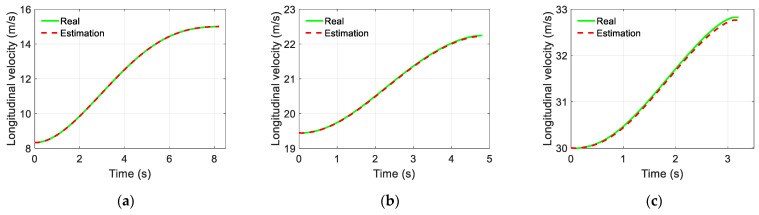
Longitudinal velocity estimated by EKF: (**a**) Scenario A; (**b**) Scenario B; (**c**) Scenario C.

**Figure 8:**
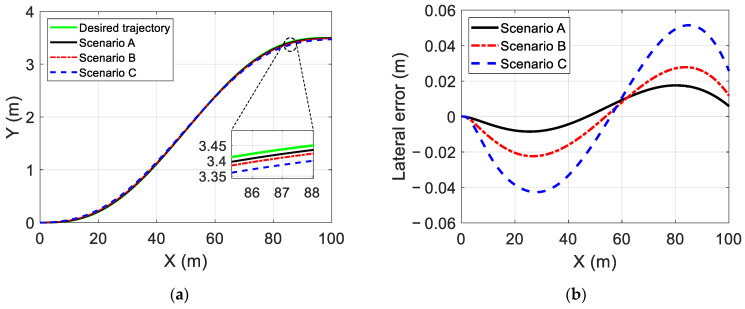
Trajectory tracking results for different scenarios and lateral tracking error: (**a**) Trajectory tracking results of the proposed controller in different scenarios; (**b**) Lateral tracking error.

The simulation results of the trajectory-tracking controller are displayed in Figure 6, Figure 7 and Figure 8. Figure 6 and Figure 7 show the estimated results of the yaw rate and longitudinal velocity, respectively, under three scenarios. Figure 6 and Figure 7 show that the estimated value is very close to the real value; there is a relatively large error in the middle of the lane change, but all the estimated errors are within a reasonable range, which means that the EKF can estimate the yaw rate and longitudinal velocity accurately. Figure 8 shows the trajectory tracking results of the coupling controller proposed in this paper, and compares them with the desired trajectory. The vehicle can effectively track the desired trajectory at different gear speeds, as shown in Figure 8a. Moreover, the lateral tracking error of the vehicle is always kept within the range of 0.06 m, and even within 0.03 m in two of the specific scenarios as show in Figure 8b.

### 5.2. Comparision of Simulation Results

**Figure 9:**
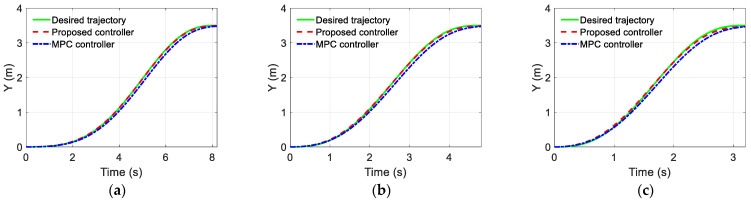
Desired trajectory tracking results comparison of proposed controller with MPC: (**a**) Scenario A; (**b**) Scenario B; (**c**) Scenario C.

**Figure 10:**
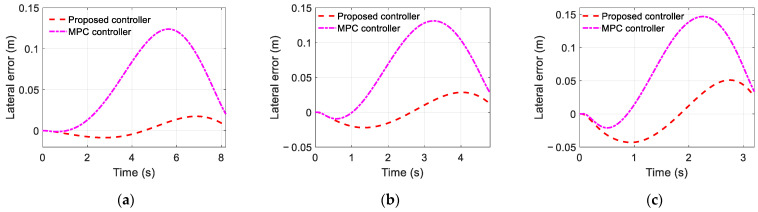
Lateral position error: (**a**) Scenario A; (**b**) Scenario B; (**c**) Scenario C.

**Figure 11:**
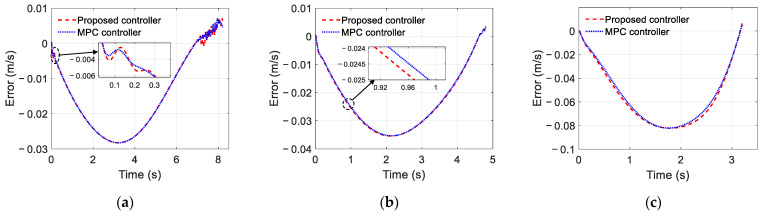
Speed tracking error: (**a**) Scenario A; (**b**) Scenario B; (**c**) Scenario C.

To further demonstrate the advantages of the proposed trajectory-tracking controller, Figure 9 and Figure 10 also show the simulation results of the trajectory tracking for the proposed controller and MPC controller under the same vehicle parameters and simulation scenarios. As shown in Figure 9, both controllers can complete the single lane change condition and enter the target lane within the planning time. However, compared with the MPC controller, the lane changing trajectory of the proposed controller is closer to the desired trajectory. Moreover, as shown in Figure 10, the maximum trajectory tracking error of the MPC controller exceeds 0.15 m, and this error can be reduced by choosing the appropriate predictive time domain and control time domain. As shown in Figure 10a, it can be kept within 0.13 m, but it is still large compared with the proposed controller. Figure 11 compares the speed-tracking errors for the two controllers. As shown in Figure 11, the performance of the speed tracking for the two approaches is similar.

To compare the trajectory-tracking accuracy of the two controllers, we provide an extensive comparison of the two controllers. The errors when reaching the end of trajectory planning are shown in Table 4. The maximum and average values of the lateral position errors are described as shown in Table 5 and Table 6, respectively.

Additionally, the RMSE performance index between the actual lateral position of the simulated vehicle and the desired trajectory was computed to further compare the trajectory-tracking accuracy of the two controllers. The results are shown in Table 7. The RMSE is formulized as follows:

(61) $$\mathrm{RMSE}=\sqrt{\frac{1}{n}\sum_{i=1}^{m}{\left(y_{i}-Y_{i}\right)}^{2}},$$

where $y_{i}$ is the actual position of the simulated vehicle in CarSim at time i, and $Y_{i}$ is the desired position at the time i.

## 6. Conclusions

This paper presented a lateral and longitudinal coupling controller for a trajectory-tracking control system while an observer based on the EKF was established to obtain the longitudinal velocity and yaw rate, which are sensitive to the trajectory-tracking control and hard to measure directly using the existing vehicle sensors. A coupling controller combining LQR and MPC was proposed to track the desired trajectory, and the controller ensured that the lateral position error and the speed tracking error were kept within a small range. The performance of the proposed controller has been verified in three different scenarios by co-simulation (CarSim-Matlab/Simulink). Finally, the simulation results were compared with the MPC controller, and the results illustrated that the proposed controller was superior to the MPC controller.

With respect to our future research, the next task is to consider the influence of axle load transfer on vehicle mass, that is, to develop an observer to estimate the vehicle mass, thereby attempting to address the problem of the mass changes in the front axle and rear axle caused by acceleration, which leads to the change in cornering stiffness.

## Figures and Tables

**Table 1:** Symbols and their descriptions.

Symbol	Description	Symbol	Description
m (kg)	vehicle weight	θ (deg)	vehicle heading angle
$l_{f}$ (m)	distance from centroid to front axle	$\theta_{r}$ (deg)	heading angle for the desired path
$l_{r}$ (m)	distance from centroid to rear axle	$a_{des}$ (m·s^2^)	desired acceleration
V (m·s^−1^)	velocity at the center of vehicle mass	a (m·s^2^)	vehicle’s actual acceleration
$v_{x}$ (m·s^−1^)	velocity components of V along X axis	χ	system state
$v_{y}/\dot{y}$ (m·s^−1^)	velocity components of V along Y axis	$u_{i}$	control input
φ	yaw angle of the vehicle	Q	state error weighting matrix for LQR
$\dot{\varphi }/\omega$ (rad·s^−1^)	yaw rate of the vehicle	R	control quantity weighting matrix for LQR
β (deg)	slip angle of the center of mass	$\delta_{f}$	feedforward control quantity
δ (deg)	front wheel steering angle	$u_{L,min}$	minimum value of acceleration
$\alpha_{f}$ (deg)	slip angle of the front tire	$u_{L,max}$	maximum value of acceleration
$\alpha_{r}$ (deg)	slip angle of the rear tire	$\Delta u_{L,min}$	minimum value of acceleration increment
$C_{af}$ (N·rad^−1^)	front wheel cornering stiffness	$\Delta u_{L,max}$	maximum value of acceleration increment
$C_{ar}$ (N·rad^−1^)	rear wheel cornering stiffness	$a_{x}$ (m·s^2^)	longitudinal acceleration of the vehicle
$F_{yf}$ (N)	lateral force of the front tire	$a_{y}$ (m·s^2^)	lateral acceleration of the vehicle
$F_{yr}$ (N)	lateral force of the rear tire	$W_{k}$	system noise
$I_{z}$ (kg·m^2^)	moment of inertia about Z axis	$V_{k}$	measurement noise

**Table 2:** Parameters for co-simulation.

Parameter	Value (Unit)	Parameter	Value (Unit)
m	1723 (kg)	$C_{ar}$	−62,700 (N·rad^−1^)
g	9.8 (m·s^−2^)	$I_{z}$	4175 (kg·m^2^)
$l_{f}$	1.232 (m)	D	100 (m)
$l_{r}$	1.468 (m)	W	3.5 (m)
$C_{af}$	−66,900 (N·rad^−1^)	f	0.85

**Table 3:** Desired simulation scenarios.

	Initial Velocity	Final Velocity	Time of Lane Change
Scenario A	30 km/h	54 km/h	8.2 s
Scenario B	70 km/h	80 km/h	4.8 s
Scenario C	108 km/h	118 km/h	3.2 s

**Table 4:** The errors when reaching the end of trajectory planning.

	Proposed Controller	MPC Controller	Improvement (Relative to MPC)
Scenario A	0.0059 m	0.0204 m	71.08%
Scenario B	0.0119 m	0.0278 m	57.19%
Scenario C	0.0257 m	0.0337 m	23.74%

**Table 5:** The maximum values of the lateral position errors.

	Proposed Controller	MPC Controller	Improvement (Relative to MPC)
Scenario A	0.0176 m	0.1240 m	85.81%
Scenario B	0.0286 m	0.1312 m	78.20%
Scenario C	0.0510 m	0.1468 m	65.26%

**Table 6:** The average values of the lateral position errors.

	Proposed Controller	MPC Controller	Improvement (Relative to MPC)
Scenario A	0.0080 m	0.0601 m	86.69%
Scenario B	0.0160 m	0.0662 m	75.83%
Scenario C	0.0302 m	0.0720 m	58.06%

**Table 7:** The RMSE between the actual lateral position of the simulated vehicle and desired trajectory.

	Proposed Controller	MPC Controller	Improvement (Relative to MPC)
Scenario A	0.0093	0.0749	87.58%
Scenario B	0.0182	0.0817	77.72%
Scenario C	0.0339	0.0910	62.75%

## Data Availability

The data presented in this study are available on request from the corresponding author.
